# (*N*-Butyl-*N*-phenyl­dithio­carbamato-κ*S*)triphenyl­tin(IV)

**DOI:** 10.1107/S1600536811012426

**Published:** 2011-04-07

**Authors:** Nurul Farahana Kamaludin, Ibrahim Baba, Normah Awang, Mohamed Ibrahim Mohamed Tahir, Edward R. T. Tiekink

**Affiliations:** a Environmental Health Programme, Faculty of Allied Health Sciences, Universiti Kebangsaan Malaysia, Jalan Raja Muda Aziz, 50300 Kuala Lumpur, Malaysia; b School of Chemical Sciences and Food Technology, Faculty of Science and Technology, Universiti Kebangsaan Malaysia, 43600 Bangi, Malaysia; c Department of Chemistry, Universiti Putra Malaysia, 43400 Serdang, Malaysia; d Department of Chemistry, University of Malaya, 50603 Kuala Lumpur, Malaysia

## Abstract

The title compound, [Sn(C_6_H_5_)_3_(C_11_H_14_NS_2_)], features a tetra­hedrally coordinated Sn atom, as the dithio­carbamate ligand coordinates in a monodentate fashion. Due to the proximity of the non-coordinating thione S atom, distortions from ideal tetra­hedral geometry about the metal atom are evident with the widest C—Sn—S angle being 117.26 (5)°. In the crystal, mol­ecules are linked by C—H⋯S inter­actions, which generate helical supra­molecular chains along the *b* axis.

## Related literature

For a review on the applications and structural chemistry of tin dithio­carbamates, see: Tiekink (2008 ▶). For a recently reported related structure, see: Awang *et al.* (2010 ▶).
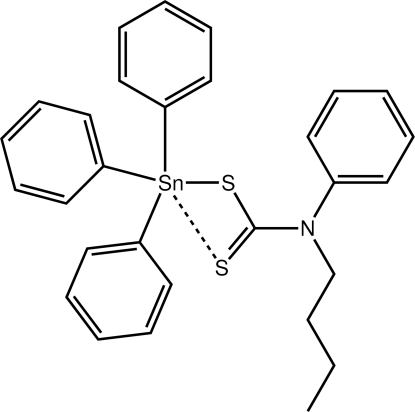


## Experimental

### 

#### Crystal data

[Sn(C_6_H_5_)_3_(C_11_H_14_NS_2_)]
*M* *_r_* = 574.34
Monoclinic, 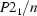

*a* = 10.0488 (1) Å
*b* = 18.0008 (2) Å
*c* = 15.2054 (2) Å
β = 102.442 (1)°
*V* = 2685.85 (5) Å^3^
*Z* = 4
Mo *K*α radiation
μ = 1.12 mm^−1^
*T* = 150 K
0.24 × 0.22 × 0.10 mm

#### Data collection

Oxford Diffraction Xcaliber Eos Gemini diffractometer
Absorption correction: multi-scan (*CrysAlis PRO*; Oxford Diffraction, 2010 ▶) *T*_min_ = 0.781, *T*_max_ = 0.894
34109 measured reflections
6099 independent reflections
5277 reflections with *I* > 2σ(*I*)
*R*_int_ = 0.044

#### Refinement

*R*[*F* ^2^ > 2σ(*F* ^2^)] = 0.024
*wR*(*F* ^2^) = 0.060
*S* = 1.02
6099 reflections
299 parameters
H-atom parameters constrained
Δρ_max_ = 0.49 e Å^−3^
Δρ_min_ = −0.27 e Å^−3^

### 

Data collection: *CrysAlis PRO* (Oxford Diffraction, 2010 ▶); cell refinement: *CrysAlis PRO*; data reduction: *CrysAlis PRO*; program(s) used to solve structure: *SHELXS97* (Sheldrick, 2008 ▶); program(s) used to refine structure: *SHELXL97* (Sheldrick, 2008 ▶); molecular graphics: *ORTEP-3* (Farrugia, 1997 ▶) and *DIAMOND* (Brandenburg, 2006 ▶); software used to prepare material for publication: *publCIF* (Westrip, 2010 ▶).

## Supplementary Material

Crystal structure: contains datablocks global, I. DOI: 10.1107/S1600536811012426/hb5836sup1.cif

Structure factors: contains datablocks I. DOI: 10.1107/S1600536811012426/hb5836Isup2.hkl

Additional supplementary materials:  crystallographic information; 3D view; checkCIF report

## Figures and Tables

**Table:** 

Sn—S1	2.4772 (5)
Sn—S2	3.1048 (5)
Sn—C12	2.1286 (18)
Sn—C18	2.1380 (19)
Sn—C24	2.1521 (18)
S1—C1	1.758 (2)
S2—C1	1.675 (2)

**Table:** 

C12—Sn—C18	113.76 (7)
C12—Sn—C24	107.51 (7)
C18—Sn—C24	107.45 (7)
C12—Sn—S1	117.26 (5)
C18—Sn—S1	115.54 (5)
C24—Sn—S1	92.18 (5)

**Table 2:** Hydrogen-bond geometry (Å, °)

*D*—H⋯*A*	*D*—H	H⋯*A*	*D*⋯*A*	*D*—H⋯*A*
C28—H28⋯S2^i^	0.95	2.83	3.612 (2)	140

Symmetry code: (i) 
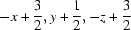
.

**Fig. 1.**
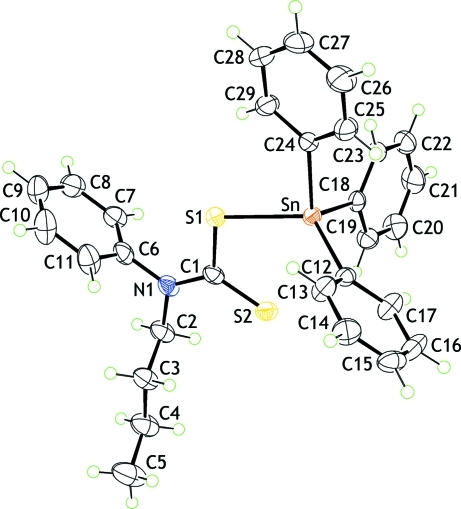
The molecular structure of (I) showing the displacement ellipsoids at the 50% probability level.

**Fig. 2.**
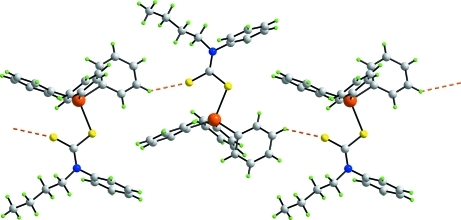
A view of the helical supramolecular chain in (I) mediated by C—H···S interactions (orange dashed lines) along the b axis in (I).

## supplementary crystallographic information

### Comment

Their potential as anti-cancer agents, anti-microbials and insecticides, and as convenient synthetic precursors for tin sulfide nanoparticles, attract continued attention to organotin dithiocarbamates (Tiekink, 2008). As part of on-going of structural studies of triphenyltin(IV) dithiocarbamates (Awang *et al.*, 2010), the analysis of the title compound, (I), was undertaken.

The molecular structure, Fig. 1, features Sn coordinated by the dithiocarbamate ligand and three *ipso*-C atoms of three benzene rings. The dithiocarbamte ligand coordinates essentially in a monodentate fashion, an assignment supported by the large disparity in the C—S bond distances, Table 1. The coordination geometry is based on a tetrahedron with the range of tetrahedral angles being 92.18 (5) to 117.26 (5) °. The wider angles are ascribed to the influence of the proximate S2 atom. The crystal packing of (I) features helical supramolecular chains along the *b* axis that is sustained by C—H···S interactions, Fig. 2 and Table 2.

### Experimental

The title compound was prepared using an *in situ* method by the addition of carbon disulfide (0.01 mol) to an ethanolic solution of *N*-butylphenylamine (0.01 mol). The mixture was stirred for 1 h at 277 K. The solution was then added drop-wise to a solution of triphenyltin(IV) chloride (0.01 mol) in ethanol (20 ml). The mixture was stirred for 1 h. The white precipitate that formed was filtered, washed with cold ethanol and dried in a desiccator. Crystallization was carried out by using an ethanol:chloroform (1:1 *v*/*v*) mixture to yield colourless prisms of (I). Yield: 34%. *M*.pt. 374–375 K. Elemental analysis. Found (calculated) for C_29_H_29_NS_2_Sn: C, 60.54 (60.64); H 5.08 (5.09); N 2.34 (2.44); S 11.31 (11.17); Sn 20.19 (20.66) %. UV (CHCl_3_) λ_max_ 252 nm (*L*(π) →*L*(π*)). IR (KBr): ν(C—H) 2960 m; ν(C≐N) 1477 m; ν(N—C) 1139 s; ν(C≐S) 997 s; ν(Cd—S) 356 s cm^-1^.

### Refinement

Carbon-bound H-atoms were placed in calculated positions (C—H 0.95 to 0.99 Å) and were included in the refinement in the riding model approximation, with *U*_iso_(H) set to 1.2 to 1.5*U*_equiv_(C).

### Crystal data

**Table:**

[Sn(C_6_H_5_)_3_(C_11_H_14_NS_2_)]	*F*(000) = 1168
*M**_r_* = 574.34	*D*_x_ = 1.420 Mg m^−^^3^
Monoclinic, *P*2_1_/*n*	Mo *K*α radiation, λ = 0.71073 Å
Hall symbol: -P 2yn	Cell parameters from 18395 reflections
*a* = 10.0488 (1) Å	θ = 2–29°
*b* = 18.0008 (2) Å	µ = 1.12 mm^−^^1^
*c* = 15.2054 (2) Å	*T* = 150 K
β = 102.442 (1)°	Prism, colourless
*V* = 2685.85 (5) Å^3^	0.24 × 0.22 × 0.10 mm
*Z* = 4	

### Data collection

**Table:**

Oxford Diffraction Xcaliber Eos Gemini diffractometer	6099 independent reflections
Radiation source: fine-focus sealed tube	5277 reflections with *I* > 2σ(*I*)
graphite	*R*_int_ = 0.044
Detector resolution: 16.1952 pixels mm^-1^	θ_max_ = 27.5°, θ_min_ = 2.2°
ω scans	*h* = −13→13
Absorption correction: multi-scan (*CrysAlis PRO*; Oxford Diffraction, 2010)	*k* = −23→23
*T*_min_ = 0.781, *T*_max_ = 0.894	*l* = −19→19
34109 measured reflections	

### Refinement

**Table:**

Refinement on *F*^2^	Primary atom site location: structure-invariant direct methods
Least-squares matrix: full	Secondary atom site location: difference Fourier map
*R*[*F*^2^ > 2σ(*F*^2^)] = 0.024	Hydrogen site location: inferred from neighbouring sites
*wR*(*F*^2^) = 0.060	H-atom parameters constrained
*S* = 1.02	*w* = 1/[σ^2^(*F*_o_^2^) + (0.0284*P*)^2^ + 0.6995*P*] where *P* = (*F*_o_^2^ + 2*F*_c_^2^)/3
6099 reflections	(Δ/σ)_max_ = 0.003
299 parameters	Δρ_max_ = 0.49 e Å^−^^3^
0 restraints	Δρ_min_ = −0.27 e Å^−^^3^

### Special details

**Table:**

Geometry. All s.u.'s (except the s.u. in the dihedral angle between two l.s. planes) are estimated using the full covariance matrix. The cell s.u.'s are taken into account individually in the estimation of s.u.'s in distances, angles and torsion angles; correlations between s.u.'s in cell parameters are only used when they are defined by crystal symmetry. An approximate (isotropic) treatment of cell s.u.'s is used for estimating s.u.'s involving l.s. planes.
Refinement. Refinement of *F*^2^ against ALL reflections. The weighted *R*-factor *wR* and goodness of fit *S* are based on *F*^2^, conventional *R*-factors *R* are based on *F*, with *F* set to zero for negative *F*^2^. The threshold expression of *F*^2^ > 2σ(*F*^2^) is used only for calculating *R*-factors(gt) *etc*. and is not relevant to the choice of reflections for refinement. *R*-factors based on *F*^2^ are statistically about twice as large as those based on *F*, and *R*- factors based on ALL data will be even larger.

### Fractional atomic coordinates and isotropic or equivalent isotropic displacement parameters (Å^2^)

**Table:**

	*x*	*y*	*z*	*U*_iso_*/*U*_eq_	
Sn	0.636843 (12)	0.514323 (7)	0.706567 (8)	0.02366 (5)	
S1	0.74819 (5)	0.56428 (3)	0.85614 (3)	0.03110 (11)	
S2	0.60687 (5)	0.42144 (3)	0.87326 (3)	0.03087 (11)	
N1	0.72072 (18)	0.50776 (9)	1.01112 (11)	0.0316 (4)	
C1	0.6917 (2)	0.49591 (10)	0.92221 (13)	0.0280 (4)	
C2	0.6806 (2)	0.45399 (12)	1.07424 (13)	0.0366 (5)	
H2A	0.5941	0.4299	1.0445	0.044*	
H2B	0.6642	0.4811	1.1276	0.044*	
C3	0.7875 (2)	0.39454 (12)	1.10512 (14)	0.0392 (5)	
H3A	0.8713	0.4180	1.1404	0.047*	
H3B	0.8106	0.3705	1.0518	0.047*	
C4	0.7378 (3)	0.33576 (13)	1.16269 (15)	0.0471 (6)	
H4A	0.6524	0.3135	1.1280	0.057*	
H4B	0.7172	0.3597	1.2168	0.057*	
C5	0.8423 (3)	0.27455 (16)	1.19190 (19)	0.0671 (8)	
H5A	0.8668	0.2525	1.1386	0.101*	
H5B	0.8035	0.2362	1.2247	0.101*	
H5C	0.9239	0.2955	1.2312	0.101*	
C6	0.7940 (2)	0.57343 (12)	1.05062 (13)	0.0338 (5)	
C7	0.7210 (2)	0.63472 (12)	1.06800 (14)	0.0365 (5)	
H7	0.6242	0.6337	1.0544	0.044*	
C8	0.7902 (3)	0.69796 (13)	1.10562 (15)	0.0442 (5)	
H8	0.7407	0.7403	1.1178	0.053*	
C9	0.9303 (3)	0.69916 (15)	1.12510 (15)	0.0518 (6)	
H9	0.9775	0.7427	1.1498	0.062*	
C10	1.0025 (3)	0.63747 (16)	1.10886 (17)	0.0578 (7)	
H10	1.0993	0.6384	1.1228	0.069*	
C11	0.9344 (2)	0.57402 (15)	1.07221 (15)	0.0485 (6)	
H11	0.9842	0.5312	1.0620	0.058*	
C12	0.71232 (19)	0.41209 (10)	0.66655 (12)	0.0258 (4)	
C13	0.8499 (2)	0.40110 (12)	0.67362 (15)	0.0377 (5)	
H13	0.9118	0.4400	0.6962	0.045*	
C14	0.8999 (2)	0.33402 (12)	0.64823 (16)	0.0442 (5)	
H14	0.9952	0.3274	0.6540	0.053*	
C15	0.8124 (3)	0.27783 (12)	0.61507 (15)	0.0421 (5)	
H15	0.8465	0.2319	0.5986	0.050*	
C16	0.6745 (3)	0.28803 (12)	0.60564 (17)	0.0498 (6)	
H16	0.6132	0.2494	0.5811	0.060*	
C17	0.6240 (2)	0.35448 (12)	0.63171 (15)	0.0404 (5)	
H17	0.5285	0.3607	0.6257	0.048*	
C18	0.41962 (19)	0.52313 (10)	0.67250 (13)	0.0263 (4)	
C19	0.3332 (2)	0.47347 (11)	0.70304 (14)	0.0329 (4)	
H19	0.3709	0.4336	0.7413	0.039*	
C20	0.1932 (2)	0.48155 (13)	0.67828 (17)	0.0439 (6)	
H20	0.1357	0.4477	0.7004	0.053*	
C21	0.1365 (2)	0.53874 (15)	0.62151 (17)	0.0489 (6)	
H21	0.0403	0.5446	0.6052	0.059*	
C22	0.2205 (2)	0.58712 (13)	0.58878 (15)	0.0426 (5)	
H22	0.1819	0.6256	0.5485	0.051*	
C23	0.3614 (2)	0.57999 (11)	0.61427 (13)	0.0328 (4)	
H23	0.4184	0.6140	0.5919	0.039*	
C24	0.71761 (18)	0.60219 (10)	0.63749 (12)	0.0260 (4)	
C25	0.7478 (2)	0.59142 (12)	0.55345 (14)	0.0388 (5)	
H25	0.7282	0.5450	0.5238	0.047*	
C26	0.8063 (3)	0.64789 (14)	0.51222 (16)	0.0510 (6)	
H26	0.8271	0.6395	0.4550	0.061*	
C27	0.8344 (2)	0.71606 (13)	0.55379 (16)	0.0472 (6)	
H27	0.8737	0.7546	0.5252	0.057*	
C28	0.8049 (3)	0.72759 (12)	0.63682 (16)	0.0455 (6)	
H28	0.8242	0.7743	0.6659	0.055*	
C29	0.7472 (2)	0.67142 (11)	0.67806 (14)	0.0383 (5)	
H29	0.7272	0.6802	0.7355	0.046*	

### Atomic displacement parameters (Å^2^)

**Table:**

	*U*^11^	*U*^22^	*U*^33^	*U*^12^	*U*^13^	*U*^23^
Sn	0.02646 (8)	0.02098 (8)	0.02380 (8)	−0.00103 (5)	0.00594 (5)	−0.00096 (5)
S1	0.0410 (3)	0.0271 (2)	0.0234 (2)	−0.0021 (2)	0.0029 (2)	0.00049 (19)
S2	0.0389 (3)	0.0250 (2)	0.0299 (2)	0.0037 (2)	0.0100 (2)	0.00173 (19)
N1	0.0396 (10)	0.0330 (9)	0.0223 (8)	0.0080 (7)	0.0072 (7)	0.0010 (7)
C1	0.0301 (10)	0.0281 (10)	0.0259 (10)	0.0106 (8)	0.0061 (8)	0.0028 (8)
C2	0.0456 (12)	0.0418 (12)	0.0253 (10)	0.0110 (10)	0.0138 (9)	0.0053 (9)
C3	0.0446 (13)	0.0421 (12)	0.0316 (11)	0.0116 (10)	0.0101 (10)	0.0074 (9)
C4	0.0654 (16)	0.0486 (14)	0.0314 (11)	0.0150 (12)	0.0196 (11)	0.0083 (10)
C5	0.087 (2)	0.0596 (17)	0.0554 (17)	0.0233 (16)	0.0181 (15)	0.0239 (14)
C6	0.0410 (12)	0.0404 (12)	0.0194 (9)	0.0069 (9)	0.0051 (8)	0.0001 (8)
C7	0.0408 (12)	0.0376 (12)	0.0317 (11)	0.0069 (9)	0.0091 (9)	0.0032 (9)
C8	0.0604 (15)	0.0376 (12)	0.0352 (12)	0.0068 (11)	0.0111 (11)	−0.0012 (10)
C9	0.0596 (16)	0.0566 (15)	0.0342 (12)	−0.0082 (13)	−0.0009 (11)	−0.0076 (11)
C10	0.0419 (14)	0.078 (2)	0.0483 (15)	0.0000 (13)	−0.0023 (12)	−0.0180 (14)
C11	0.0415 (13)	0.0615 (16)	0.0389 (13)	0.0146 (11)	0.0005 (10)	−0.0115 (11)
C12	0.0340 (10)	0.0218 (9)	0.0230 (9)	−0.0014 (8)	0.0090 (8)	0.0004 (7)
C13	0.0342 (11)	0.0316 (11)	0.0468 (13)	−0.0021 (9)	0.0076 (10)	−0.0081 (9)
C14	0.0435 (13)	0.0396 (12)	0.0501 (14)	0.0115 (10)	0.0111 (11)	−0.0039 (11)
C15	0.0688 (16)	0.0272 (11)	0.0359 (12)	0.0091 (11)	0.0238 (11)	0.0014 (9)
C16	0.0684 (17)	0.0299 (12)	0.0554 (15)	−0.0169 (11)	0.0226 (13)	−0.0156 (11)
C17	0.0415 (12)	0.0348 (12)	0.0482 (13)	−0.0096 (9)	0.0171 (10)	−0.0120 (10)
C18	0.0270 (9)	0.0280 (10)	0.0234 (9)	0.0008 (7)	0.0046 (8)	−0.0057 (7)
C19	0.0310 (10)	0.0334 (11)	0.0344 (11)	−0.0019 (8)	0.0073 (9)	0.0015 (9)
C20	0.0322 (11)	0.0515 (14)	0.0491 (14)	−0.0063 (10)	0.0111 (10)	−0.0004 (11)
C21	0.0295 (11)	0.0597 (15)	0.0526 (15)	0.0049 (11)	−0.0017 (11)	−0.0041 (12)
C22	0.0413 (12)	0.0402 (12)	0.0402 (12)	0.0093 (10)	−0.0045 (10)	−0.0001 (10)
C23	0.0393 (11)	0.0291 (10)	0.0276 (10)	−0.0006 (9)	0.0023 (9)	−0.0013 (8)
C24	0.0265 (9)	0.0249 (9)	0.0253 (9)	−0.0006 (7)	0.0025 (8)	0.0026 (7)
C25	0.0540 (14)	0.0325 (11)	0.0312 (11)	−0.0070 (10)	0.0122 (10)	−0.0023 (9)
C26	0.0723 (17)	0.0504 (15)	0.0358 (12)	−0.0063 (13)	0.0237 (12)	0.0062 (11)
C27	0.0547 (14)	0.0398 (13)	0.0481 (14)	−0.0105 (11)	0.0131 (12)	0.0156 (11)
C28	0.0639 (15)	0.0261 (11)	0.0432 (13)	−0.0108 (10)	0.0046 (12)	0.0037 (9)
C29	0.0588 (14)	0.0265 (10)	0.0308 (11)	−0.0036 (10)	0.0122 (10)	−0.0013 (8)

### Geometric parameters (Å, °)

**Table:**

Sn—S1	2.4772 (5)	C12—C13	1.377 (3)
Sn—S2	3.1048 (5)	C12—C17	1.393 (3)
Sn—C12	2.1286 (18)	C13—C14	1.394 (3)
Sn—C18	2.1380 (19)	C13—H13	0.9500
Sn—C24	2.1521 (18)	C14—C15	1.364 (3)
S1—C1	1.758 (2)	C14—H14	0.9500
S2—C1	1.675 (2)	C15—C16	1.374 (3)
N1—C1	1.337 (2)	C15—H15	0.9500
N1—C6	1.453 (3)	C16—C17	1.390 (3)
N1—C2	1.479 (3)	C16—H16	0.9500
C2—C3	1.517 (3)	C17—H17	0.9500
C2—H2A	0.9900	C18—C19	1.394 (3)
C2—H2B	0.9900	C18—C23	1.396 (3)
C3—C4	1.524 (3)	C19—C20	1.384 (3)
C3—H3A	0.9900	C19—H19	0.9500
C3—H3B	0.9900	C20—C21	1.385 (3)
C4—C5	1.521 (3)	C20—H20	0.9500
C4—H4A	0.9900	C21—C22	1.378 (3)
C4—H4B	0.9900	C21—H21	0.9500
C5—H5A	0.9800	C22—C23	1.391 (3)
C5—H5B	0.9800	C22—H22	0.9500
C5—H5C	0.9800	C23—H23	0.9500
C6—C11	1.378 (3)	C24—C25	1.389 (3)
C6—C7	1.382 (3)	C24—C29	1.394 (3)
C7—C8	1.391 (3)	C25—C26	1.390 (3)
C7—H7	0.9500	C25—H25	0.9500
C8—C9	1.375 (3)	C26—C27	1.381 (3)
C8—H8	0.9500	C26—H26	0.9500
C9—C10	1.377 (4)	C27—C28	1.373 (3)
C9—H9	0.9500	C27—H27	0.9500
C10—C11	1.385 (4)	C28—C29	1.382 (3)
C10—H10	0.9500	C28—H28	0.9500
C11—H11	0.9500	C29—H29	0.9500
			
C12—Sn—C18	113.76 (7)	C13—C12—C17	118.00 (18)
C12—Sn—C24	107.51 (7)	C13—C12—Sn	121.01 (14)
C18—Sn—C24	107.45 (7)	C17—C12—Sn	120.99 (15)
C12—Sn—S1	117.26 (5)	C12—C13—C14	121.2 (2)
C18—Sn—S1	115.54 (5)	C12—C13—H13	119.4
C24—Sn—S1	92.18 (5)	C14—C13—H13	119.4
C1—S1—Sn	97.64 (7)	C15—C14—C13	120.1 (2)
C1—N1—C6	121.91 (17)	C15—C14—H14	119.9
C1—N1—C2	121.41 (18)	C13—C14—H14	119.9
C6—N1—C2	116.68 (16)	C16—C15—C14	119.7 (2)
N1—C1—S2	123.79 (16)	C16—C15—H15	120.2
N1—C1—S1	116.04 (15)	C14—C15—H15	120.2
S2—C1—S1	120.16 (12)	C15—C16—C17	120.5 (2)
N1—C2—C3	112.88 (17)	C15—C16—H16	119.8
N1—C2—H2A	109.0	C17—C16—H16	119.8
C3—C2—H2A	109.0	C16—C17—C12	120.5 (2)
N1—C2—H2B	109.0	C16—C17—H17	119.8
C3—C2—H2B	109.0	C12—C17—H17	119.8
H2A—C2—H2B	107.8	C19—C18—C23	118.40 (18)
C2—C3—C4	111.70 (18)	C19—C18—Sn	123.25 (14)
C2—C3—H3A	109.3	C23—C18—Sn	118.31 (14)
C4—C3—H3A	109.3	C20—C19—C18	120.8 (2)
C2—C3—H3B	109.3	C20—C19—H19	119.6
C4—C3—H3B	109.3	C18—C19—H19	119.6
H3A—C3—H3B	107.9	C19—C20—C21	120.4 (2)
C3—C4—C5	112.3 (2)	C19—C20—H20	119.8
C3—C4—H4A	109.1	C21—C20—H20	119.8
C5—C4—H4A	109.1	C22—C21—C20	119.5 (2)
C3—C4—H4B	109.1	C22—C21—H21	120.2
C5—C4—H4B	109.1	C20—C21—H21	120.2
H4A—C4—H4B	107.9	C21—C22—C23	120.5 (2)
C4—C5—H5A	109.5	C21—C22—H22	119.8
C4—C5—H5B	109.5	C23—C22—H22	119.8
H5A—C5—H5B	109.5	C22—C23—C18	120.4 (2)
C4—C5—H5C	109.5	C22—C23—H23	119.8
H5A—C5—H5C	109.5	C18—C23—H23	119.8
H5B—C5—H5C	109.5	C25—C24—C29	117.65 (18)
C11—C6—C7	120.5 (2)	C25—C24—Sn	121.76 (14)
C11—C6—N1	120.44 (19)	C29—C24—Sn	120.53 (14)
C7—C6—N1	119.07 (19)	C24—C25—C26	120.7 (2)
C6—C7—C8	119.5 (2)	C24—C25—H25	119.7
C6—C7—H7	120.2	C26—C25—H25	119.7
C8—C7—H7	120.2	C27—C26—C25	120.5 (2)
C9—C8—C7	120.0 (2)	C27—C26—H26	119.7
C9—C8—H8	120.0	C25—C26—H26	119.7
C7—C8—H8	120.0	C28—C27—C26	119.5 (2)
C8—C9—C10	120.2 (2)	C28—C27—H27	120.3
C8—C9—H9	119.9	C26—C27—H27	120.3
C10—C9—H9	119.9	C29—C28—C27	120.1 (2)
C9—C10—C11	120.2 (2)	C29—C28—H28	120.0
C9—C10—H10	119.9	C27—C28—H28	120.0
C11—C10—H10	119.9	C28—C29—C24	121.6 (2)
C6—C11—C10	119.6 (2)	C28—C29—H29	119.2
C6—C11—H11	120.2	C24—C29—H29	119.2
C10—C11—H11	120.2		
			
C12—Sn—S1—C1	−68.43 (8)	C13—C14—C15—C16	0.9 (4)
C18—Sn—S1—C1	70.03 (8)	C14—C15—C16—C17	−1.6 (4)
C24—Sn—S1—C1	−179.43 (8)	C15—C16—C17—C12	1.0 (4)
C6—N1—C1—S2	−179.67 (14)	C13—C12—C17—C16	0.4 (3)
C2—N1—C1—S2	0.9 (3)	Sn—C12—C17—C16	179.86 (17)
C6—N1—C1—S1	0.3 (2)	C12—Sn—C18—C19	58.98 (18)
C2—N1—C1—S1	−179.14 (14)	C24—Sn—C18—C19	177.86 (16)
Sn—S1—C1—N1	−173.43 (14)	S1—Sn—C18—C19	−80.93 (16)
Sn—S1—C1—S2	6.51 (12)	C12—Sn—C18—C23	−118.75 (14)
C1—N1—C2—C3	88.8 (2)	C24—Sn—C18—C23	0.13 (16)
C6—N1—C2—C3	−90.7 (2)	S1—Sn—C18—C23	101.34 (14)
N1—C2—C3—C4	−174.46 (18)	C23—C18—C19—C20	−1.8 (3)
C2—C3—C4—C5	178.3 (2)	Sn—C18—C19—C20	−179.52 (16)
C1—N1—C6—C11	−87.1 (2)	C18—C19—C20—C21	0.9 (4)
C2—N1—C6—C11	92.3 (2)	C19—C20—C21—C22	0.8 (4)
C1—N1—C6—C7	94.2 (2)	C20—C21—C22—C23	−1.7 (4)
C2—N1—C6—C7	−86.3 (2)	C21—C22—C23—C18	0.8 (3)
C11—C6—C7—C8	1.5 (3)	C19—C18—C23—C22	0.9 (3)
N1—C6—C7—C8	−179.84 (18)	Sn—C18—C23—C22	178.78 (15)
C6—C7—C8—C9	0.1 (3)	C12—Sn—C24—C25	29.77 (18)
C7—C8—C9—C10	−1.1 (4)	C18—Sn—C24—C25	−93.05 (17)
C8—C9—C10—C11	0.5 (4)	S1—Sn—C24—C25	149.29 (16)
C7—C6—C11—C10	−2.1 (3)	C12—Sn—C24—C29	−147.32 (16)
N1—C6—C11—C10	179.2 (2)	C18—Sn—C24—C29	89.86 (17)
C9—C10—C11—C6	1.1 (4)	S1—Sn—C24—C29	−27.81 (16)
C18—Sn—C12—C13	172.10 (15)	C29—C24—C25—C26	0.5 (3)
C24—Sn—C12—C13	53.25 (18)	Sn—C24—C25—C26	−176.71 (18)
S1—Sn—C12—C13	−48.72 (17)	C24—C25—C26—C27	−0.6 (4)
C18—Sn—C12—C17	−7.36 (19)	C25—C26—C27—C28	0.5 (4)
C24—Sn—C12—C17	−126.21 (16)	C26—C27—C28—C29	−0.2 (4)
S1—Sn—C12—C17	131.82 (15)	C27—C28—C29—C24	0.1 (4)
C17—C12—C13—C14	−1.1 (3)	C25—C24—C29—C28	−0.2 (3)
Sn—C12—C13—C14	179.44 (17)	Sn—C24—C29—C28	177.00 (17)
C12—C13—C14—C15	0.5 (4)		

### Hydrogen-bond geometry (Å, °)

**Table:**

*D*—H···*A*	*D*—H	H···*A*	*D*···*A*	*D*—H···*A*
C28—H28···S2^i^	0.95	2.83	3.612 (2)	140

Symmetry codes: (i) −*x*+3/2, *y*+1/2, −*z*+3/2.
